# CircNT5E promotes the proliferation and migration of bladder cancer via sponging miR-502-5p

**DOI:** 10.7150/jca.53385

**Published:** 2021-03-01

**Authors:** Jinhui Yang, Xiaoyun Liu, Guangcheng Dai, Lanying Qu, Bo Tan, Bo Zhu, Fuming Qi, Xinyu Gai, Bo Cheng

**Affiliations:** 1 Urology and Andrology Department, Shengli OilFiled Central Hospital, Dongying, 257034, Shandong, China.; 2 Department of Urology, The Second Affiliated Hospital of Soochow University, 215004, Suzhou, China.

**Keywords:** Bladder cancer, circNT5E, NT5E, miR-502-5p, HOXC8.

## Abstract

Accumulating evidence suggest that circRNA RNAs (circRNAs) play important roles in tumor formation and development. circNT5E has been shown to be an oncogenic gene in several types of cancer, and the high expression of circNT5E lead to tumorigenesis and cancer progression. However, the precise role of circNT5E in bladder cancer (Bca) has not been characterized. In this study, we observed that circNT5E expression was augmented in Bca tissues compared with that in adjacent normal tissues, and its expression level was positively associated with larger tumor size and lower survival rate. Further experiments showed that suppression of circNT5E restrained the growth and metastasis of Bca cells *in vitro*. circNT5E was mainly distributed in the cytoplasm and it captured miR-502-5p to increase HOXC8 mRNA and protein expression. Moreover, decreased miR-502-5p obviously reversed the circNT5E silencing-mediated inhibition of Bca cell growth and migration. Thus, this study suggested that circNT5E may act as a pro-oncogene in the development and progression of Bca and it may become a useful tumor biomarker and promising therapeutic target for Bca treatment.

## Introduction

Bladder cancer (BCa) is the most common malignant cancer in the urinary system with high morbidity and mortality, bringing huge economic burden 1. As has been estimated, there are 549,393 new Bca cases and 199,992 cancer deaths worldwide in 2018 2. Approximately a quarter of Bca are diagnosed as non-muscle invasive Bca (NMIBC), whereas the rest are classified as muscle-invasive BC (MIBC) 3. More than one half MIBC patients progresses to distant metastasis with extremely low 5-year survival rate and poor prognosis 4, 5. Lacking of deep insight of the biological and molecular mechanisms of Bca initiation and development cause the current Bca treatment dilemma. Hence, there is an urgent need to investigate critical molecular signal pathways and therapeutic target to fight against Bca.

Circular RNA (circRNA), a class of single-stranded closed RNA molecules, is formed by the splice of the precursor mRNA 6, 7. Unlike liner RNA, circRNA is created by the reverse splice of 5' and 3' end of the precursor mRNA to form closed ring structure 8. CircRNA is widely expressed in human organs, tissues and cells 9, getting involved in complex cellular life activities, such as cell differentiation, proliferation, senescence and apoptosis 10-13. An ocean of circRNAs are abnormally expressed in tissues and cells under pathological conditions, which can lead to the occurrence and development of various diseases, such as cardiovascular diseases, diabetes and tumors 14-17. A previous study showed that circNT5E could sponge he suppressor miRNA miR-442a to promote the growth and metastasis of glioblastoma cells and inhibit cell apoptosis *in vitro* and *in vivo* 18. In non-small cell lung cancer, circNT5E could accelerate the growth and metastasis of lung cancer cells and inhibit cell apoptosis via suppressing miR-134 expression 19. However, the biological and pathological functions of circNT5E has not been characterized and require further investigation.

In this study, we discovered that circNT5E expression was augmented in Bca tissues compared with that in adjacent normal tissues, and its expression level was positively associated with larger tumor size and lower survival rate. Further experiments showed that suppression of circNT5E restrained the growth and metastasis of Bca cells *in vitro*. circNT5E was mainly distributed in the cytoplasm and it captured miR-502-5p to increase HOXC8 mRNA and protein expression. Moreover, decreased miR-502-5p obviously reversed the circNT5E silencing-mediated inhibition of Bca cell growth and migration. To sup up, this study suggested that circNT5E may act as a pro-oncogene in the development and progression of Bca and it may become a useful tumor biomarker and promising therapeutic target for Bca treatment.

## Material and methods

### Patient samples

51 Bca tissues and matched adjacent normal bladder tissues were collected from Bca patients who received surgery. This study was approved by the Ethical Committee of Shengli Oilfield Central Hospital before samples collection. Written informed consent was signed by each Bca patients included in this study. American Joint Committee on Cancer Classification Criteria was used to distinguish the clinicopathological classification and stage of Bca.

### Cell lines

SV-HUC1 and human Bca cell lines (5637, T24 and SW780) were provided from the American Type Culture Collection (ATCC). SV-HUC1 cell was grown in F12K medium (Gibco, Carlsbad, USA) supplemented with 1% penicillin/streptomycin and 10% fetal bovine serum (FBS) (Gibco, South America). Bca cells were cultured in DMEM medium mixed with 1% penicillin/streptomycin and 10% FBS. All cells included in this study were cultured in a humid incubator containing 5% CO_2_ at 37°C.

### Cell transfection

The siRNA targeting on circNT5E (sicircNT5E) and negative control (shiNC) were synthesized GenePharma (Suzhou, China). miRNA mimics and inhibitors were provided by RiboBio (Guangzhou, China). The sequences include in this study are provided in Supplementary Table 1. The sequence of circNT5E and coding sequence (CDS) of HOXC8 were cloned into the vector pcDNA3.1, respectively. Cell transfection were carried out by using Lipofectamine 3000 reagent (Invitrogen, USA).

### Cell proliferation assay

Cell counting Kit-8 (CCK-8) and clone formation assays were performed to detect the proliferation of Bca cells. For clone formation assay, the transfected Bca cells were seeded into 6-well plates for 14 days. Then, cells were fixed with 4% paraformaldehyde and stained with 0.1% crystal violet. The colonies were imaged by using a microscope and washed with 33% glacial acetic acid. For CCK-8 assay, the transfected Bca cells were cultured in 96-well plates. The OD value of Bca cells were measured by using a microplate reader at 450nm after 0, 24, 48 and 72 hours.

### Wound-healing assay

The transfected Bca cells were seeded into 6-well plates. Then, the wound field was produced by using a yellow tip until cells reached to 90%-100% confluence. The injured cells were cultured in DMEM medium without FBS for 24 hours. Finally, the migrated cells were washed by PBS and then microscopically observed and photographed at 5× magnification.

### RNA extraction and quantitative real-time PCR (qRT-PCR)

The extraction of total RNA from Bca tissue samples and cell lines was performed by using TRIzol reagent (Life technology, USA). RNA from the nuclear and cytoplasmic fraction of Bca cells was extracted by using NE-PER Nuclear and Cytoplasmic Extraction Reagents (Thermo Scientific, USA). qRT-PCR assay was performed by using a standard SYBR Green PCR Kit (Takara, Japan), and this reaction was conducted by using an ABI PRISM 7500 Fluorescent Quantitative PCR instrument. GAPDH or U6 were served as the internal standard control. The sequence of all primers included in this study were shown in Table S1. Gene expression level was measured by 2^-ΔΔCT^ method.

### RNase R digestion

RNase R kit (Epicenter, WI, USA) was used to examine the stability of circRNA. Total RNA and RNase R kit were incubated at 37 °C for 30 min according to the manufacturer's instrument. Finally, qRT-PCR assay was conducted to access the expression level of circNT5E and NT5E upon RNase R digestion.

### RNA pull-down assay

Biotin-coupled probe RNA pull down assay was used to detect the miRNA bounded with circNT5E. The sequence of the biotin-coupled circNE5E probe was shown in Supplement Table 1. Bca cells overexpressing circNT5E were lysed and incubated with the specific circNT5E probe at 37°C for 16 hours. The biotin-coupled RNA complex was extracted, reversed and placed through qRT-PCR assay.

### Luciferase reporter assay

Dual-luciferase reporter assay was carried out by using a luciferase reporter kit (Promega, USA) in accordance with the manufacturer's protocol. circNE5E-Wild Type (WT)/ Mutant (Mut) and HOXC8 WT/Mut reporter plasmids were constructed and co-transfected into SW780 and T24 cells along with miR-502-5p mimics using Lipofectamine 3000. After 48 h, the luciferase activities were calculated utilizing a microplate reader.

### Western blotting assay

Total protein from the Bca transfected cells were extracted using RIPA lysis buffer (Beyotime, Shanghai) mixed with protease inhibitor (Beyotime, Shanghai). Equivalent amount of protein was separated on 6-10% SDS/PAGE gels and transferred onto PVDF membranes. After blocking membranes with 5% skim milk powder, the membranes were incubated with primary at 4 °C for 16 h, followed by a secondary antibody incubation. Finally, the blots were visualized using a BioSpectrum 600 Imaging System (UVP, CA, USA).

### *In vivo* assay

The tumor xenograft transplantation assay was reviewed and approved by the ethical review committee of Shengli Oilfield Central Hospital. 4-week old BALB/cNude mice were randomly assigned to siNC group and sicicrcNT5E group (n=5 for each group). Approximately 6× 10^6^ SW780 cells infected with sicircNT5E lentiviral vector or negative control vector were subcutaneously injected into the back of mice. The of transplantation tumors were measured every 8 days. Finally, all mice were euthanized and the tumors were peeled off and weighted after 40 days.

### Statistical analyses

All statistical analysis was conducted by using Graphpad Prism 7 and SPSS 20.0 (IBM, Chicago, USA). Student's t-test or ANOVA test were used to analyzed the differences between groups. Kaplan-Meier test was applied to evaluate the overall survival curves.

A P value less that 0.05 was regarded as statistically significant.

## Results

### CircNT5E is overexpressed in Bca tissues and expression is positively associated with unfavorable prognosis

**Figure 1:**
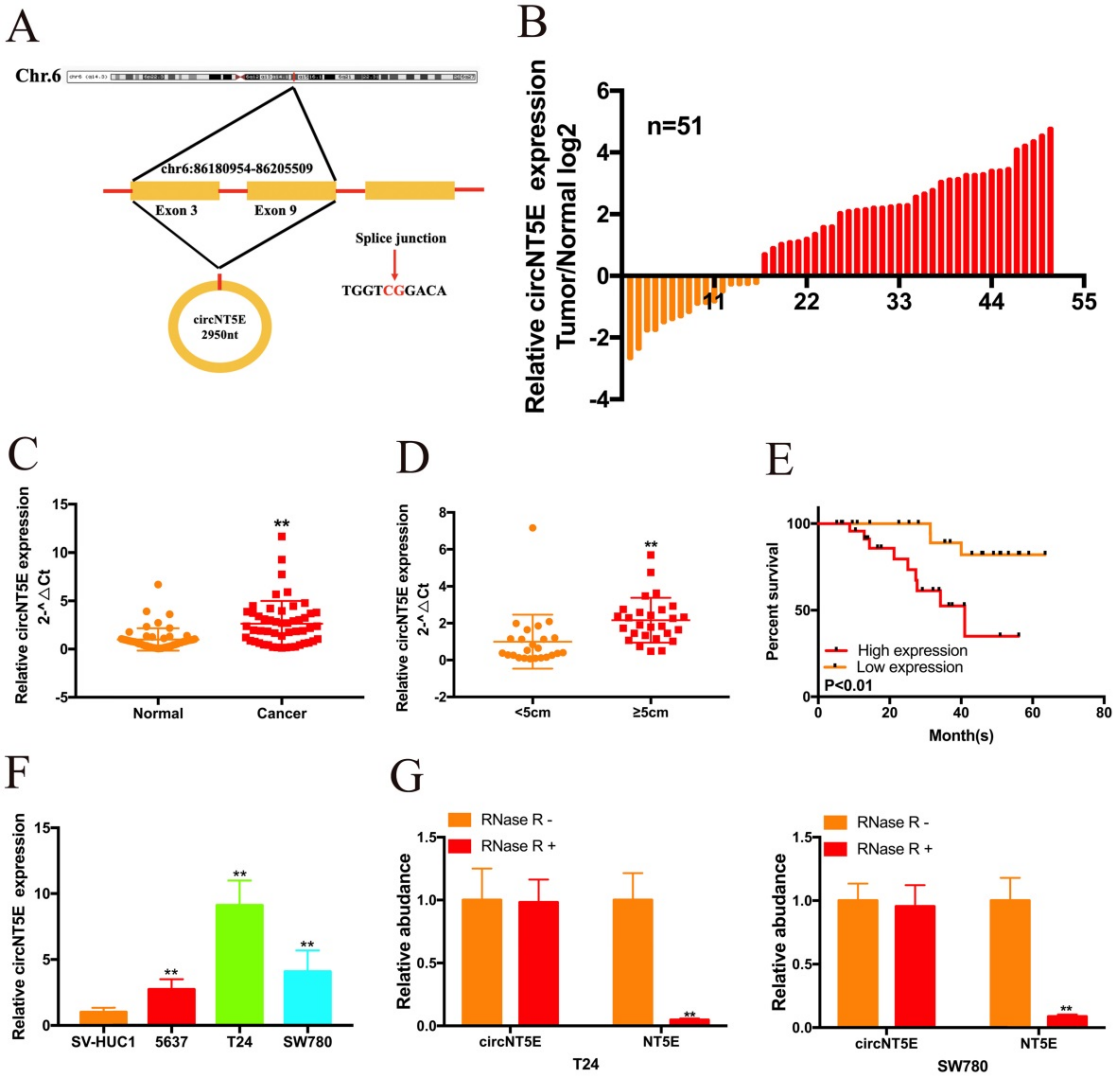
circNT5E expression is significantly augmented in Bca tissues and positively corelated with unfavorable prognosis. (A) The schematic of circNT5E formation. (B and C) The expression level of circNT5E in Bca tissues and non-tumor tissues is presented. (D) The expression level of circNT5E in different tumor size of Bca tissues. (E) The overall survival rate of Bca patients with low and high circNT5E expression. (F) circNT5E expression was obviously increased in Bca cells compared to that in SV-HUC1 cell. (G) qRT-PCR assay was carried out to detect the ability of circNT5E and NT5E to tolerate the digestion of RNase R. *P <0.05, **P<0.01 and NS: none significant.

By searching the Circbase Database, we discovered that circNT5E is located on chr3:123471177-123512691. CicrNT5E is derived from back-splicing of exons of 3 to 10 of TLK1 and formed a 2950 nt circular transcript (Figure 1A). qRT-PCR assay was carried out to measure circNT5E expression in 51 Bca tissues and matched normal tissues. As presented in Figure 1B and C, circNT5E expression was significantly augmented in in 68.6% (35 of 51) of Bca tissues. Orange column represents relative low expression of circNT5E, while red column represents relative increased expression of circNT5E. In addition, increased expression of circNT5E was positively correlated with bigger tumor size of Bca tissues (Figure 1D and Table 1). Compared to low circNT5E expression, Bca patients with high circNT5E expression levels had a lower overall survival rate (Figure 1E). Compared with SV-HUC1, circNT5E expression was significantly overexpressed in 5637, T24 and SW780 cells. T24 and SW780 cells were selected as the objective for further experiments (Figure 1F). Finally, the stability of circNT5E was detected by RNase R digestion, and the results showed that NT5E mRNA expression decreased obviously after RNase R digestion, but RNase R failed to digest circNT5E (Figure 1G).

### Suppression of circNT5E significantly impaired the proliferation and migration of Bca cells

**Figure 2:**
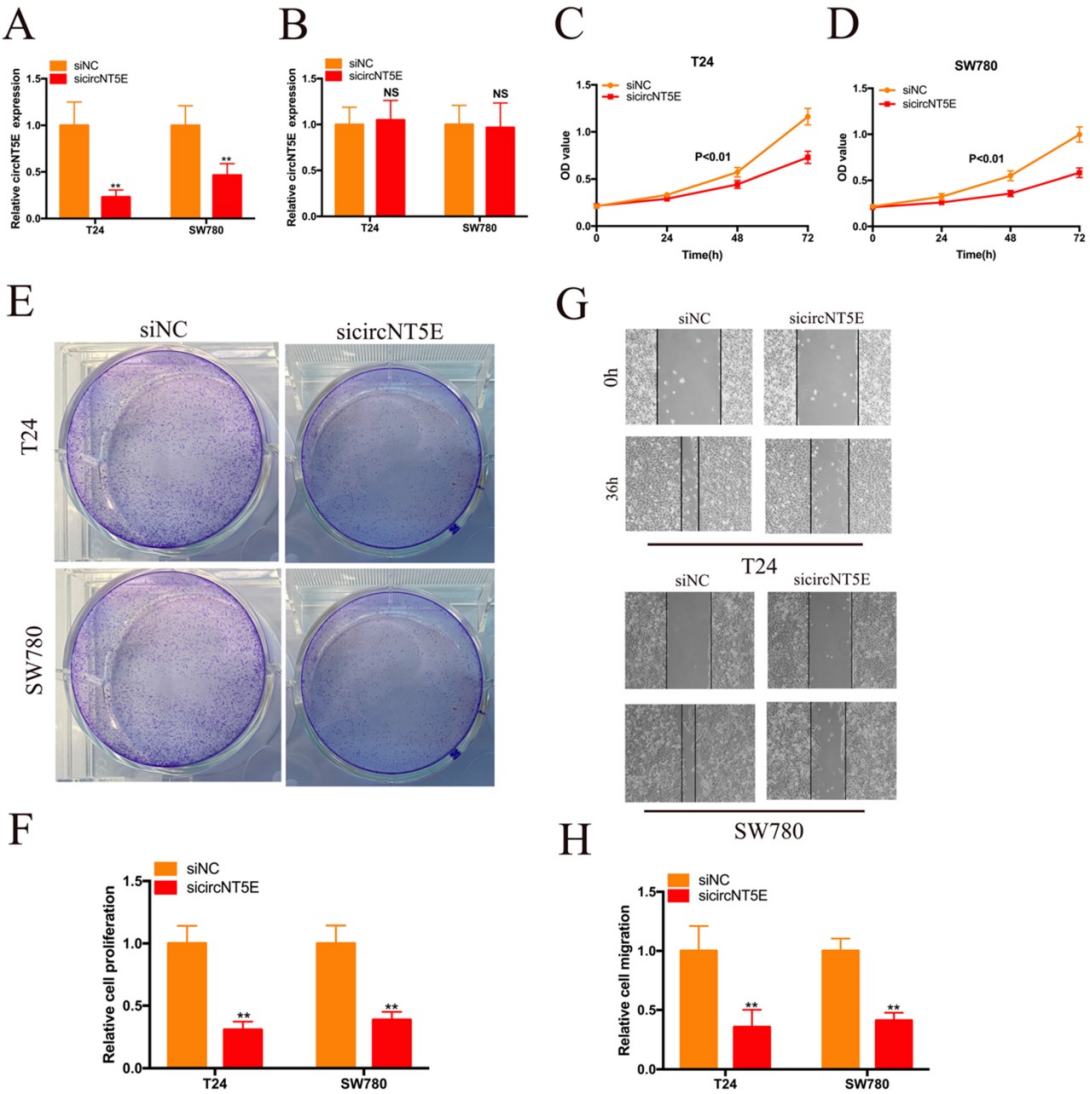
Decreased expression of circNT5E impaired the proliferation and migration of T24 and SW780 cells. (A, B) qRT-PCR assay was conducted the evaluate the circNT5E and NT5E in Bca cells transfected with siRNA targeting circNT5E. (C, D) The CCK-8 results showed the growth cure of Bca cells transfected with sicircNT5E. (E, F) The colony formation assays showed the colony formation ability of Bca cells transfected with sicircNT5E. (G, H) Wound-healing assay showed the migrated ability of Bca cells transfected with sicircNT5E. *P <0.05, **P<0.01 and NS: none significant.

To detect the biological role of circNT5E in Bca cells, a siRNA targeting circNT5E (sicircNT5E) was applied to inhibit circNT5E in Bca cells. As shown in Figure 2A, circNT5E expression was significantly suppressed by sicircNT5E in Bca cells. However, knockdown of circNT5E did not modulate NE5E mRNA expression (Figure 2B). The results of CCK-8 assay showed that silence of circNT5E significantly slow the growth of T24 and SW780 cells (Figure 2C-D). The results of colony formation assay demonstrated that silence of circNT5E notably decreased the cloning capabilities of Bca cells (Figure 2E-F). In addition, the wound-healing assay confirmed that attenuated expression of circNT5E notably impaired the migration of T24 and SW780 cells (Figure 2G-H). These results demonstrated that circNT5E could promote Bca proliferation and migration *in vitro*.

### cirNT5E abundantly sponges miR-502-5p in Bca cells

**Figure 3:**
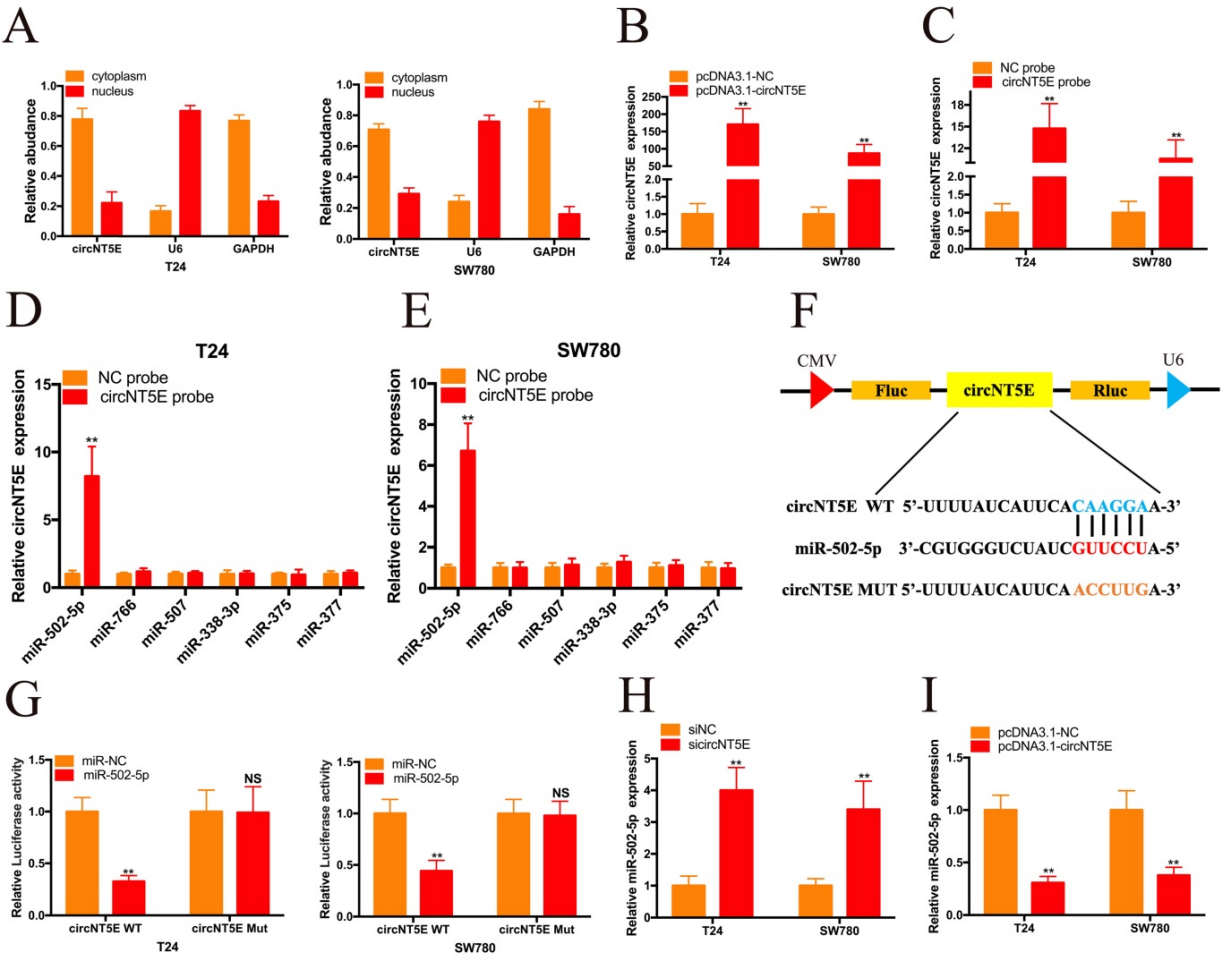
circNT5E sponges miR-502-5p in Bca cells. (A) Nucleo-cytoplasmic separation assay showed that circNT5E is mostly distributed in the cytoplasm. (B) circNT5E expression in T24 and SW780 cells transfected with pcNDA3.1-circNT5E. (C) qRT-PCR assay presented that circNT5E expression was augmented in Bca cells incubated with a specific circNT5E probe. (D, E) Only miR-502-5p was notably pulled down by a specific circNT5E probe in T24 and SW780 cells. (F, G) The luciferase activities of T24 and SW780 cells transfected with miR-502-5p mimics and luciferase reporter vectors. (H) miR-502-5p expression in T24 and SW780 cells transfected with sicircNT5E. (I) miR-502-5p expression in T24 and SW780 cells transfected with pcDNA3.1-circNT5E. *P <0.05, **P<0.01 and NS: none significant.

To investigate the molecular mechanism underlying circNT5E, we carried out nuclear mass separation assay to examine the circNT5E subcellular localization in T24 and SW780 cells. As shown in Figure 3A, circNT5E is predominantly located in the cytoplasm, suggesting that circNT5E may serve as a “miRNA sponge” to absorb miRNAs to exert its biological function. To confirm our hypothesis, we utilized Circular RNA Interactome database to forecasted the potential miRNAs bound with circNT5E. Six potential binding miRNAs were picked out, including miR-502-5p, miR-766, miR-507, miR-338-3p, miR-375 and miR-377. RNA pull-down assay was performed to investigate whether circNT5E could directly absorb these miRNAs. pcDNA3.1-circNT5E was utilized to enhance circNT5E expression in Bca cells. circNT5E expression in Bca cells was augmented dramatically after transfection of pcDNA3.1-circNT5E plasmid. The biotinylated circNT5E probe obviously pulled down circNT5E in T24 and SW780 cells after transfection of pcDNA3.1-circNT5E. The RNA pull-down assay results demonstrated only miR-502-5p was successfully pulled down by the biotinylated circNT5E probe in T24 and SW780 cells. The dual luciferase reporter assay results also confirmed that circNT5E could directly sponge miR-502-5p (Figure 3F and G). Furthermore, suppression of circNT5E significantly up-regulated the expression of miR-502-5p while forced expression of circNT5E inhibited miR-502-5p expression in Bca cells. These results suggested that circNT5E acted as a miRNA sponge for miR-502-5p.

### miR-502-5p directly binds to HOXC8

**Figure 4:**
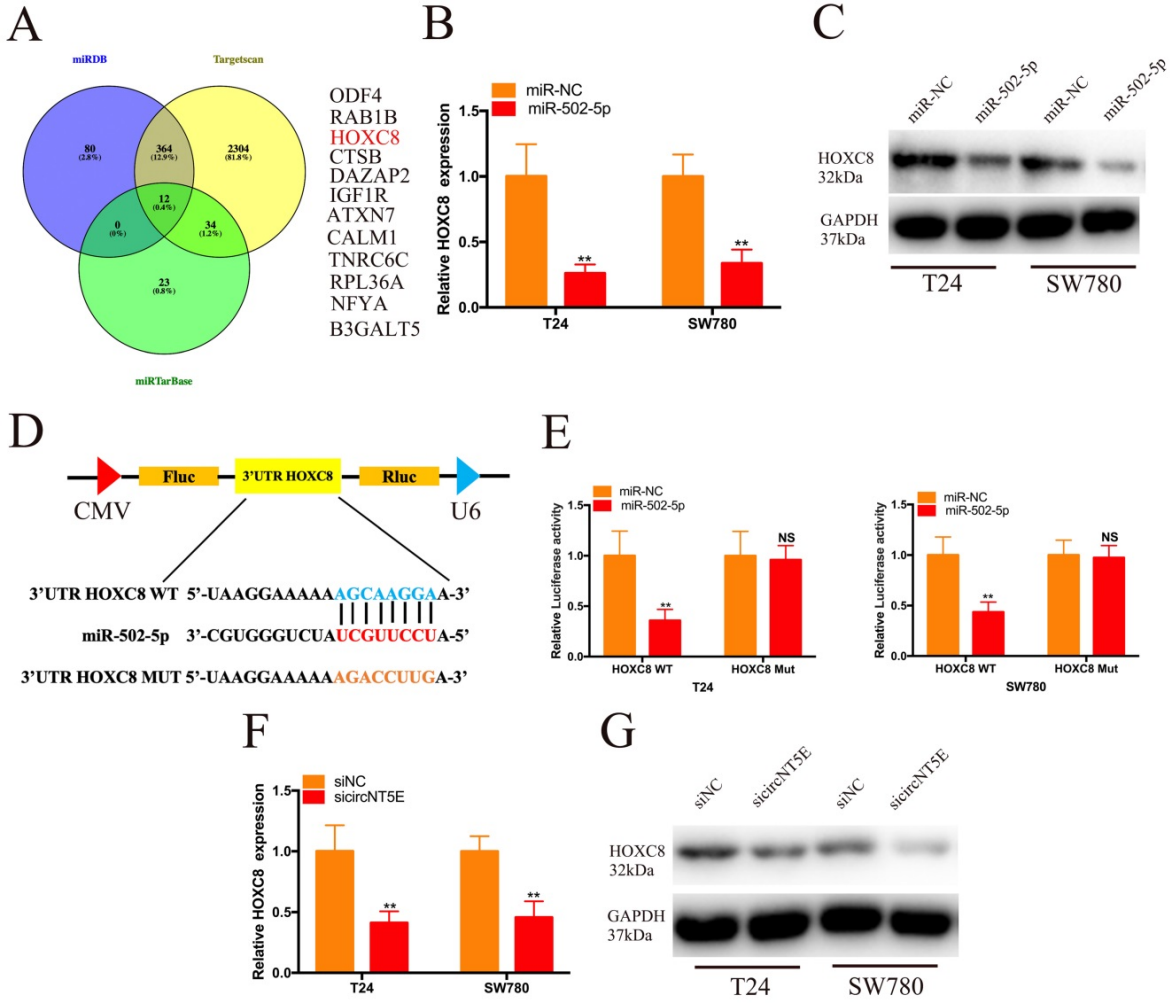
miR-502-5p directly binds to the 3'UTR of HOXC8. (A) 12 potential genes interacted with miR-502-5p were predicted by using miRDB, TargetScan and miRTarbase database. (B, C) The expression level of HOXC8 mRNA and protein in T24 and SW780 cells transfected with miR-502-5p mimics. (D, E) The luciferase activities of T24 and SW780 cells transfected with miR-502-5p mimics and luciferase reporter vectors. (F, G) The expression level of HOXC8 mRNA and protein in T24 and SW780 cells transfected with sicircNT5E. *P <0.05, **P<0.01 and NS: none significant.

To investigate the molecular mechanism of miR-502-5p involved in the progression of Bca, we combined the TargetScan, miRDB and miRTarBase to forecast the target genes of miR-502-5p and found 12 potential candidate genes, including ODF4, RAB1B, HOXC8, CTSB, DAZAP2, IGF1R, ATXN7, ATXN7, TNRC6C, TNRC6C, NFYA and B3GALT5 (Figure 4A). Then, we discovered that increased expression of miR-502-5p significantly inhibited the mRNA and protein expression of HOXC8 (Figure 4B and C). The luciferase reporter assay results demonstrated that miR-502-5p directly bound with the 3'UTR of HOXC8 (Figure 4D and E). Moreover, suppression of circNT5E restrained the HOXC8 mRNA and protein expression (Figure 4F and G).

### Enhanced expression of HOXC8 reverses the proliferation and migration inhibition of T24 and SW780 cells induced by suppressing circNT5E

**Figure 5:**
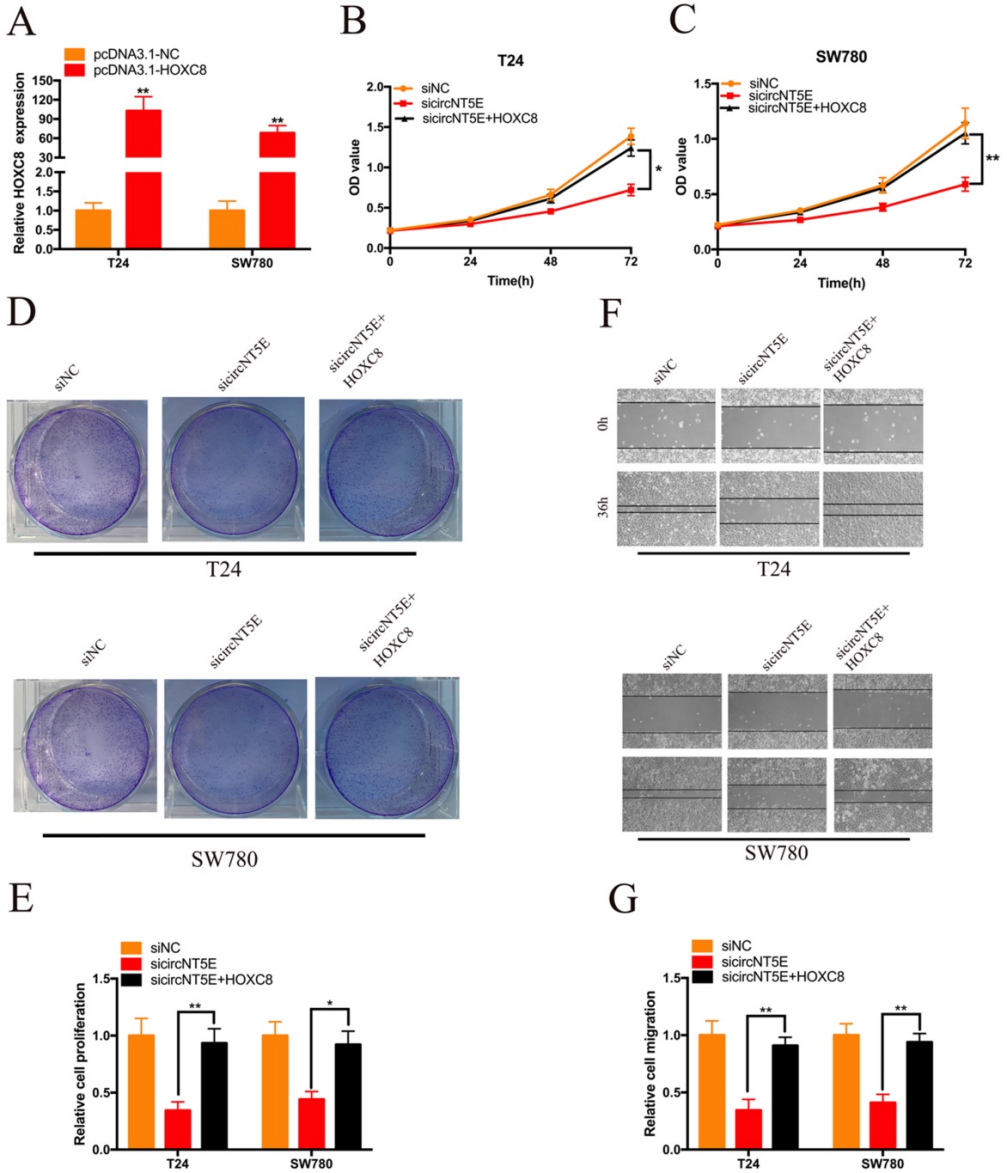
Enhanced expression of HOXC8 reverses the proliferation and migration inhibition of T24 and SW780 cells induced by suppressing circNT5E. (A) The expression level of HOXC8 mRNA in T24 and SW780 cells transfected with pcDNA3.1-HOXC8. (B-E) The results of CCK-8 and colon formation assay showed suppression of circNT5E notably impaired Bca cell proliferation, and the suppressive phenomenon was rescued after cells were co-transfected with pcDNA3.1-HOXC8. (F, G) Cell migration assay showed that suppression of circNT5E notably impaired Bca cell migration, and the suppressive phenomenon was rescued after cells were co-transfected with pcDNA3.1-HOXC8. *P < 0.05, **P < 0.01 and NS: none significant.

Next, we examined whether circNT5E promoted the proliferation and migration of T24 and SW780 cells via interacting with HOXC8. pcDNA3.1-HOXC8 was applied to improve HOXC8 expression in Bca cells. As shown in Figure 5A, HOXC8 expression in Bca cells was significantly increased after transfection of pcDNA3.1-HOXC8 (Figure 5A). The results of CCK-8 assay showed that forced expression of HOXC8 significantly rescued cell growth suppression of T24 and SW780 cells produced by silencing circNT5E (Figure 5B and C). The results of clone formation assay demonstrated that forced expression of HOXC8 significantly rescued cell proliferation suppression of T24 and SW780 cells produced by silencing circNT5E (Figure 5D and E). Finally, Wound-healing assay showed that enhanced expression of HOXC8 obviously rescued cell migration inhibition of T24 and SW780 cells produced by suppressing circNT5E expression (Figure 5F and G).

### Suppression of circNT5E restrained the growth of Bca cells *in vivo*

**Figure 6:**
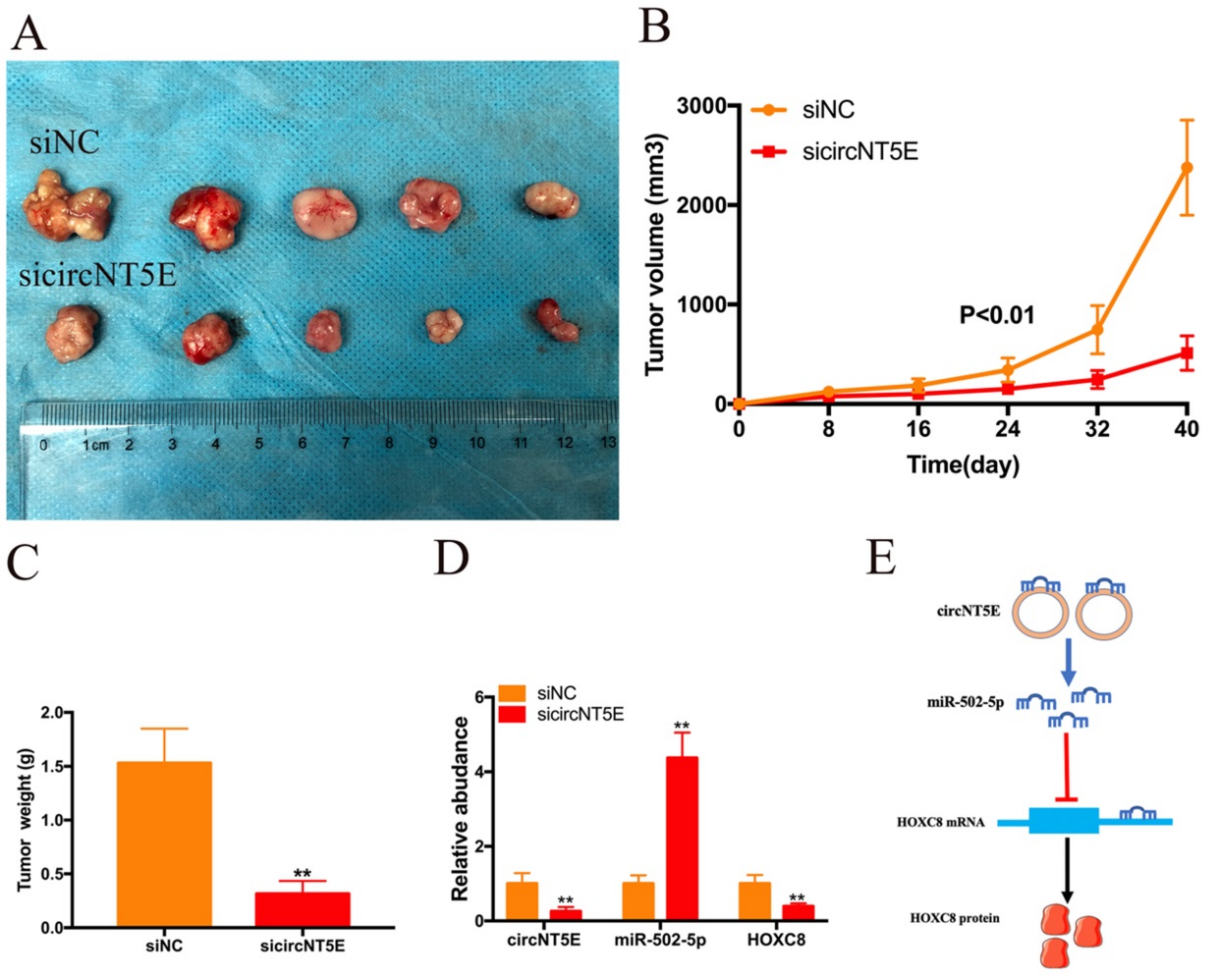
Depletion of circNT5E notably weaken the ability of Bca growth *in vivo*. (A) Subcutaneous tumor model demonstrated that the size of subcutaneous tumors derived from circNT5E depletion cells were obviously less than that collected from the negative control cells. (B) The size of subcutaneous tumors was calculated every 8 days. (C) The weight of subcutaneous tumors was evaluated after the mice were euthanized. (D) Depletion of circNT5E restrained HOXC8 expression and facilitated miR-502-5p expression *in vivo*. (E) The schematic of circNT5E/miR-502-5p/HOXC8 signal pathway. *P < 0.05, **P < 0.01 and NS: none significant.

To investigate the biological functions of circNT5E *in vivo*, SW780 cells infected with sicircNT5E lentiviral vector were injected into the back of nude mice. The subcutaneous transplanted tumors derived from circNT5E-deficient cells were smaller than those from negative group (Figure. 6A). As expected, lesser size and weight in subcutaneous transplanted tumors derived from circNT5E-deficient cells were observed compared to those tumors from negative group (Figure 6B and C). Furthermore, knockdown of circNT5E could up-regulate miR-502-5p and inhibit HOXC expression *in vivo* (Figure 6D). These data demonstrated that suppression of circNT5E restrained the growth of Bca cells *in vivo*.

## Discussion

With the rapid development of RNA research technology, a mass of circRNAs are found to be widely expressed in various kinds of cells in mammals 20, 21. Numerous studies suggest that circRNAs exert an important role in cellular life processes, such as acting as miRNA molecular sponge, regulating transcription, translating proteins and binding proteins 22-24. Abnormal expression of circRNAs can lead to the disorder of cell life activities and even tumor formation. Currently, circRNAs are being deeply studied in high incidence malignancies, such as lung cancer, breast cancer and gastric cancer 25-27. However, the research of circRNAs in bladder cancer is still in its infancy. Thus, it is urgent to deepen the study of circRNA in Bca and enrich the theoretical mechanism of the pathogenesis and metastasis of Bca.

This is the first study to detect the biological function and molecular mechanisms of circNT5E in the development of Bca. In our work, we presented that circNT5E was notably up-regulated Bca tissues. Clinical correlation analyze has shown positive correlation between circNT5E expression and the tumor size of Bca. Moreover, high expression of circNT5E cause lower survival rate of Bca patients. Further experiments showed that knockdown of circNT5E impaired the ability of Bca proliferation and migration. These results reveal that circNT5E is involved in Bca development and progression.

To explore the molecular mechanism of circNT5E promoting Bca progression, we detect the subcellular location of circNT5E and discovered that circNT5E was mostly located in the cytoplasm. Moreover, circNT5E could sponge miR-502-5p to increase HOXC8 expression (Figure 6E). Previous studies demonstrated that miR-502-5p acted as a tumor suppressor in the progression of various cancer. In gastric cancer, miR-502-5p inhibited the growth and metastasis of gastric cancer cells *in vitro* and *in vivo* by blocking the NRAS/MEK1/ERK1/2 signal pathway 28. In renal cell carcinoma (RCC), miR-502-5p impaired the proliferation and metastasis of RCC cells via decreasing SLC39A14 expression 29. In Bca, miR-502-5p restrained cell proliferation and migration *in vitro* and *in vivo* via interacting with CCND1, NOP14 and DNMT3B 30. In our study, we showed that miR-502-5p could bind to the 3'UTR of HOXC8. Previous studies suggest that HOCX8 is overexpressed in various tumors and promotes the growth and metastasis of tumor cells *in vitro* and *in vivo* 31, 32. In non-small cell lung cancer, HOXC8 acted as the transcriptional activator to strengthen TGFβ1 expression, promoting cell growth and metastasis 33. In this study, we discovered that enhanced expression of HOXC8 reverses the proliferation and migration inhibition of Bca cells induced by suppressing circNT5E, indicating that HOXC8 is a functional target gene of circNT5E.

Taken together, our data reveal that circNT5E may act as a pro-oncogene in the development and progression of Bca and it may become a useful tumor biomarker and promising therapeutic target for Bca treatment. The novel regulatory signal pathway circNT5E/miR-502-5p/HOXC8 provide a novel insight into Bca development and progression.

## Supplementary Material

Supplementary table S1.

## Figures and Tables

**Table 1:** The correlation between circNT5E expression and the clinicopathological characteristics of 51 bladder cancer patients. *P < 0.05 or **P < 0.01 was considered significant.

Parameters	Group	Total	circNT5E expression		P value
			High	Low	
Gender	Male	31	20	11	0.497
	Female	20	11	9	
Age (years)	<60	22	14	8	0.716
	≥60	29	17	12	
Tumor size (cm)	<5cm	24	11	13	0.039
	≥5cm	27	20	7	
Histological grade	low	41	27	14	0.133
	high	10	4	6	
T stage	1/2	36	18	18	0.015
	3/4	15	13	2	
N stage	N0	49	30	19	0.75
	N1-2	2	1	1	
M stage	M0	50	31	19	0.209
	M1	1	0	1	

## References

[1] Dy GW, Gore JL, Forouzanfar MH, Naghavi M, Fitzmaurice C (2017). Global Burden of Urologic Cancers, 1990-2013. Eur Urol.

[2] Bray F, Ferlay J, Soerjomataram I, Siegel RL, Torre LA, Jemal A (2018). Global cancer statistics 2018: GLOBOCAN estimates of incidence and mortality worldwide for 36 cancers in 185 countries. CA Cancer J Clin.

[3] Knowles MA (2008). Molecular pathogenesis of bladder cancer. Int J Clin Oncol.

[4] van Rhijn BW, Burger M, Lotan Y, Solsona E, Stief CG, Sylvester RJ (2009). Recurrence and progression of disease in non-muscle-invasive bladder cancer: from epidemiology to treatment strategy. Eur Urol.

[5] Alfred Witjes J, Lebret T, Comperat EM, Cowan NC, De Santis M, Bruins HM (2017). Updated 2016 EAU Guidelines on Muscle-invasive and Metastatic Bladder Cancer. Eur Urol.

[6] Memczak S, Jens M, Elefsinioti A, Torti F, Krueger J, Rybak A (2013). Circular RNAs are a large class of animal RNAs with regulatory potency. Nature.

[7] Lasda E, Parker R (2014). Circular RNAs: diversity of form and function. RNA.

[8] Li X, Yang L, Chen LL (2018). The Biogenesis, Functions, and Challenges of Circular RNAs. Mol Cell.

[9] Rybak-Wolf A, Stottmeister C, Glazar P, Jens M, Pino N, Giusti S (2015). Circular RNAs in the Mammalian Brain Are Highly Abundant, Conserved, and Dynamically Expressed. Mol Cell.

[10] Li J, Huang C, Zou Y, Ye J, Yu J, Gui Y (2020). CircTLK1 promotes the proliferation and metastasis of renal cell carcinoma by sponging miR-136-5p. Mol Cancer.

[11] Chia W, Liu J, Huang YG, Zhang C (2020). A circular RNA derived from DAB1 promotes cell proliferation and osteogenic differentiation of BMSCs via RBPJ/DAB1 axis. Cell Death Dis.

[12] Huang T, Song C, Zheng L, Xia L, Li Y, Zhou Y (2019). The roles of extracellular vesicles in gastric cancer development, microenvironment, anti-cancer drug resistance, and therapy. Mol Cancer.

[13] Xu JY, Chang NB, Rong ZH, Li T, Xiao L, Yao QP (2019). circDiaph3 regulates rat vascular smooth muscle cell differentiation, proliferation, and migration. FASEB J.

[14] Li M, Ding W, Sun T, Tariq MA, Xu T, Li P (2018). Biogenesis of circular RNAs and their roles in cardiovascular development and pathology. FEBS J.

[15] Shan K, Liu C, Liu BH, Chen X, Dong R, Liu X (2017). Circular Noncoding RNA HIPK3 Mediates Retinal Vascular Dysfunction in Diabetes Mellitus. Circulation.

[16] Li J, Huang C, Zou Y, Yu J, Gui Y (2020). Circular RNA MYLK promotes tumour growth and metastasis via modulating miR-513a-5p/VEGFC signalling in renal cell carcinoma. J Cell Mol Med.

[17] Cui X, Wang J, Guo Z, Li M, Li M, Liu S (2018). Emerging function and potential diagnostic value of circular RNAs in cancer. Mol Cancer.

[18] Wang R, Zhang S, Chen X, Li N, Li J, Jia R (2018). CircNT5E Acts as a Sponge of miR-422a to Promote Glioblastoma Tumorigenesis. Cancer Res.

[19] Dong L, Zheng J, Gao Y, Zhou X, Song W, Huang J (2020). The circular RNA NT5E promotes non-small cell lung cancer cell growth via sponging microRNA-134. Aging (Albany NY).

[20] Servick K (2017). Circular RNAs hint at new realm of genetics. Science.

[21] Nicot C (2019). RNA-Seq reveal the circular RNAs landscape of lung cancer. Mol Cancer.

[22] Kristensen LS, Andersen MS, Stagsted LVW, Ebbesen KK, Hansen TB, Kjems J (2019). The biogenesis, biology and characterization of circular RNAs. Nat Rev Genet.

[23] Zhong Y, Du Y, Yang X, Mo Y, Fan C, Xiong F (2018). Circular RNAs function as ceRNAs to regulate and control human cancer progression. Mol Cancer.

[24] Su M, Xiao Y, Ma J, Tang Y, Tian B, Zhang Y (2019). Circular RNAs in Cancer: emerging functions in hallmarks, stemness, resistance and roles as potential biomarkers. Mol Cancer.

[25] Hu W, Bi ZY, Chen ZL, Liu C, Li LL, Zhang F (2018). Emerging landscape of circular RNAs in lung cancer. Cancer Lett.

[26] Jahani S, Nazeri E, Majidzadeh AK, Jahani M, Esmaeili R (2020). Circular RNA; a new biomarker for breast cancer: A systematic review. J Cell Physiol.

[27] Shan C, Zhang Y, Hao X, Gao J, Chen X, Wang K (2019). Biogenesis, functions and clinical significance of circRNAs in gastric cancer. Mol Cancer.

[28] Zhang J, Hou L, Liang R, Chen X, Zhang R, Chen W (2019). CircDLST promotes the tumorigenesis and metastasis of gastric cancer by sponging miR-502-5p and activating the NRAS/MEK1/ERK1/2 signaling. Mol Cancer.

[29] Zeng J, Feng Q, Wang Y, Xie G, Li Y, Yang Y (2020). Circular RNA circ_001842 plays an oncogenic role in renal cell carcinoma by disrupting microRNA-502-5p-mediated inhibition of SLC39A14. J Cell Mol Med.

[30] Ying Y, Li J, Xie H, Yan H, Jin K, He L (2020). CCND1, NOP14 and DNMT3B are involved in miR-502-5p-mediated inhibition of cell migration and proliferation in bladder cancer. Cell Prolif.

[31] Gong C, Zou J, Zhang M, Zhang J, Xu S, Zhu S (2019). Upregulation of MGP by HOXC8 promotes the proliferation, migration, and EMT processes of triple-negative breast cancer. Mol Carcinog.

[32] Wang AH, Tan P, Zhuang Y, Zhang XT, Yu ZB, Li LN (2019). Down-regulation of long non-coding RNA HOTAIR inhibits invasion and migration of oesophageal cancer cells via up-regulation of microRNA-204. J Cell Mol Med.

[33] Liu H, Zhang M, Xu S, Zhang J, Zou J, Yang C (2018). HOXC8 promotes proliferation and migration through transcriptional up-regulation of TGFbeta1 in non-small cell lung cancer. Oncogenesis.

