# CircNT5E promotes the proliferation and migration of bladder cancer via sponging miR-502-5p: Erratum

**DOI:** 10.7150/jca.93985

**Published:** 2024-04-11

**Authors:** Jinhui Yang, Xiaoyun Liu, Guangcheng Dai, Lanying Qu, Bo Tan, Bo Zhu, Fuming Qi, Xinyu Gai, Bo Cheng

**Affiliations:** 1 Urology and Andrology Department, Shengli OilFiled Central Hospital, Dongying, 257034, Shandong, China.; 2 Department of Urology, The Second Affiliated Hospital of Soochow University, 215004, Suzhou, China.

After publication of the article, two problems were identified in Figure 2E and Figure 5D. We observed that the results of clone formation assay in SW780 cell were identical to that in T24 cell in Figure 2E and Figure 5D. We carefully rechecked the original images and discovered that the images of SW780 cell were misplaced in Figure 2E and Figure 5D.

All authors apologize for these errors and state that these do not change the scientific conclusions of the article. Correct images in Figure 2E and Figure 5D are provided as below.

## Figures and Tables

**Figure 2E:**
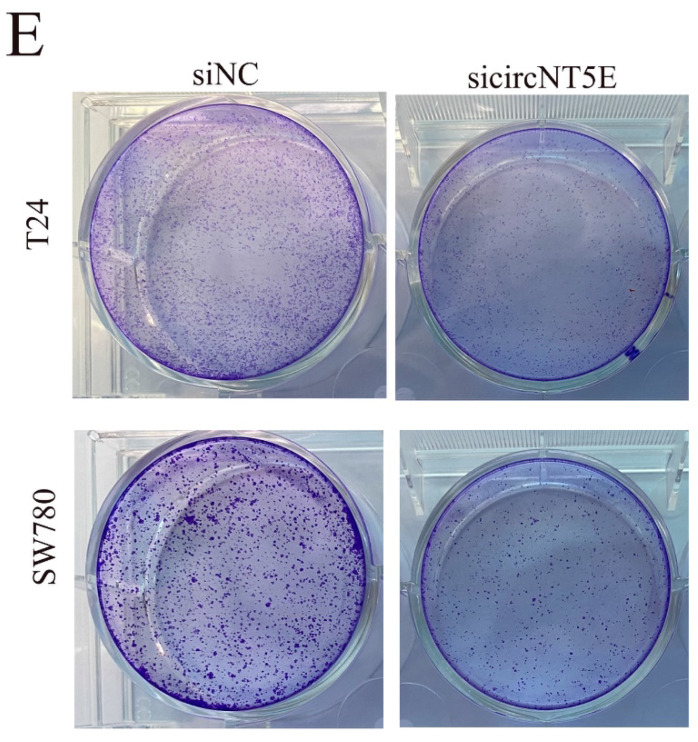
Correct image.

**Figure 5D:**
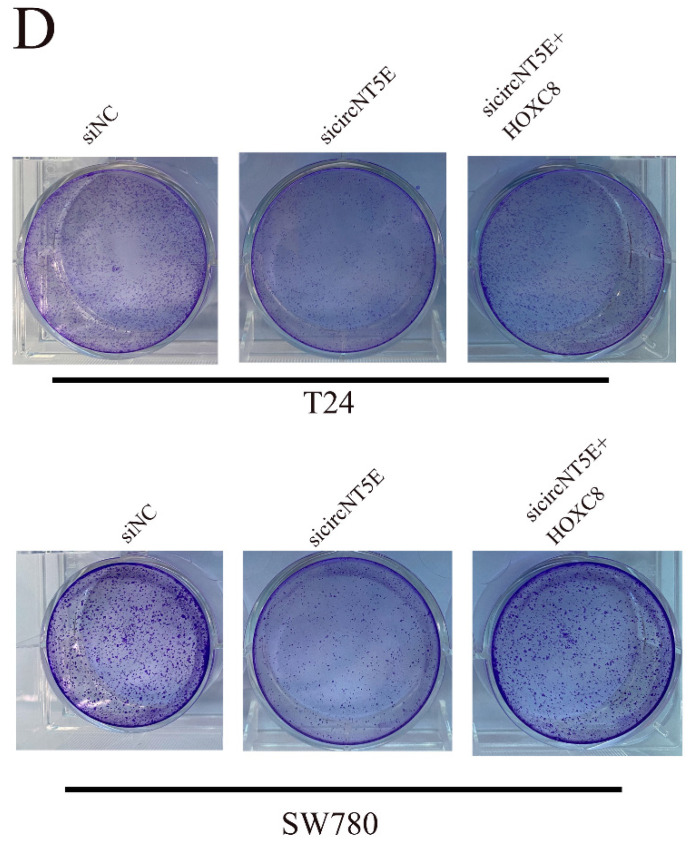
Correct image.

