# Supplementary material for: CircNT5E promotes the proliferation and migration of bladder cancer via sponging miR-502-5p
Source: J Cancer. 2021 Mar 1;12(8):2430–9. doi: 10.7150/jca.53385 (PMC7974885; doi:10.7150/jca.53385)
Supplement: Supplementary file 1 — Supplementary table S1. [file jcav12p2430s1.pdf]

Supplementary Table 1 Primers and RNA sequences used in this study

**List of oligonucleotide sequences**	**5'--> 3'**
**primers for Real-time PCR and RT-PCR**	
circNT5E-F	AGATAAGCTCTTTGGTCGGA
circNT5E-R	CGAATGTCCCAGTGCAATAA
NT5E-F	GAGGACACTCCAACACATTT
NT5E-R	TTAGAAGAATGGGATTTCCA
GAPDH-F	AATGGGCAGCCGTTAGGAAA
GAPDH-R	AATGGGCAGCCGTTAGGAAA
U6-F	TGCGGGTGCTCGCTTCGGCAGC
U6-R	CCAGTGCAGGGTCCGAGGT
HOXC8-F	TCCCTGGAACCGGCCTATTA
HOXC8-R	GCGCCTCGTAGCCATAGAAT
hsa-miR-502-5p_F:	GCGCAGATCCTTGCTATC
hsa-miR-766_F:	GACTCCAGCCCCACA
hsa-miR-507_F:	GCATTTTGCACCTTTTGGA
has-miR-338-3p_F:	GCAGTCCAGCATCAGTG
has-miR-375_F:	GAGCCCCTCGCACA
has-miR-377_F:	GCAGATCACACAAAGGCA
siRNA	
sicircNT5	CTCTTTGGTCGGACAAATTTA
RNA pull-down probe	
circNT5E	CTTCAAACACTAAATTTGTCCGACCAAAGAGC TTATCTTCAA
